# Fruit and vegetable consumption and mental health across adolescence: evidence from a diverse urban British cohort study

**DOI:** 10.1186/s12966-019-0780-y

**Published:** 2019-02-08

**Authors:** Peiyuan Huang, Majella O’Keeffe, Christelle Elia, Alexis Karamanos, Louise M. Goff, Maria Maynard, J. Kennedy Cruickshank, Seeromanie Harding

**Affiliations:** 1 Department of Nutrition Science, School of Life Course Sciences, Faculty of Life Sciences & Medicine, King’s College London, Franklin Wilkins Building, 150 Stamford Street, London, SE1 9NH UK; 2 Division of Birth Cohort Study, Guangzhou Women and Children’s Medical Center, Guangzhou Medical University, Guangzhou, China; 3 Department Epidemiology and Health, ESRC International Centre for Lifecourse Studies in Society and Health, University College London, 1-19 Torrington Place, London, WC1E 7HB UK; 4 School of Clinical & Applied Sciences, Leeds Beckett University, CL 1014 Calverley Building, City Campus, Leeds, LS1 3HE UK; 5 Department of Primary Care and Public Health, School of Population Health Sciences, Faculty of Life Sciences & Medicine, King’s College London, Addison House, Guy’s, London, SE1 1UL UK

**Keywords:** Adolescent, Mental health, Fruit and vegetables, Diet, Family context

## Abstract

**Background:**

Evidence on the relationship between fruit and vegetable consumption (FV) and mental health in adolescence is sparse and inconsistent. Social determinants of FV include ethnicity, family environments and economic disadvantage. We investigated the relationship between FV and mental health in the British multi-ethnic Determinants of Adolescents (now young Adult) Social well-being and Health (DASH) longitudinal study.

**Methods:**

A longitudinal study of 4683 adolescents living in London at age 11–13 years and followed up at 14–16 years. FV was measured using validated questions on the number of portions consumed daily. Mental health was measured by the Strengths and Difficulties Questionnaire as mean Total Difficulties Score (TDS) and by classification as a ‘probable clinical case’ (TDS > 17). Social measures included ethnicity, parenting and socioeconomic circumstances. Multilevel modelling was used to investigate the association between FV and mental health throughout adolescence.

**Results:**

Low FV was common among adolescents, with approximately 60–70% of adolescents reporting < 5 portions/day and 20–30% reporting < 1 portion/day. In late adolescence, most ethnic minority groups reported lower FV than their White peers. In fully adjusted models, < 1 portion/day remained a significant correlate with mean TDS (Coef: 0.55, 0.29–0.81, *P* < 0.001) and TDS > 17 (Odds Ratio: 1.43, 1.11–1.85, *P* = 0.007). Gender- or ethnic-specific effects were not observed. Low parental care partly attenuated the association between FV and mental health.

**Conclusions:**

Low FV is a longitudinal correlate of poor mental health across adolescence. A focus on FV in parenting interventions could yield interrelated benefits across developmental outcomes given its importance to both physical and socioemotional health.

**Electronic supplementary material:**

The online version of this article (10.1186/s12966-019-0780-y) contains supplementary material, which is available to authorized users.

## Background

Child and adolescent mental health is a global priority, with mental disorders affecting 10–20% of children and adolescents worldwide [1]. In the United Kingdom (UK), one in ten children and adolescents aged 5–16 years has clinically diagnosed mental disorders, and minority ethnic children (especially Indians) tend to have a lower rate of mental disorders compared with White children [2]. About 50% of individuals with lifetime mental health problems first experience their symptoms by the age of 14 years [3], and poor mental health is closely related to other health and development concerns in young people, notably lower educational achievements, substance abuse, violence, and poor reproductive and sexual health [4].

Low fruit and vegetable consumption (FV) is a recognised modifiable risk factor that is contributing to the rising global burden of non-communicable diseases [5]. There is strong evidence of a protective effect of FV against hypertension, coronary heart disease and stroke [6]. It is also associated with reduced cardiovascular and all-cause mortality [7]. Increasing attention is being paid to the link between dietary patterns and brain health. Population-based studies have shown that higher intake of fish, fruit and vegetables is associated with lower incidence of mood disorders [8], and a recent systematic review in children and adolescents highlighted the association between healthy dietary patterns and lower levels of depression [9]. Most studies with young people focused on effects of the whole diet (e.g. diet quality/dietary patterns) and not on food components. Although dietary pattern studies take into account the interactions between different foods and nutrients, studies focusing on individual food groups (e.g. FV) offer the opportunity to identify the role of specific foods. This may help to better identify specific components in the whole diet that are effective and identify their optimal intake, which may help inform good dietary practice as well as identifying candidate foods for further mechanistic studies. It is suggested that nutrients contained in fruit and vegetables, such as complex carbohydrates, B vitamins, antioxidants and minerals, may benefit psychological health [10]. However, the few epidemiological studies with a focus on FV showed mixed results. For example, McMartin et al. found no associations between FV at the age of 10–11 years and subsequent internalizing disorders in Canadian youth [11]. Similarly, in a prospective study of Australian adolescents, there were no associations between changes in FV from age 13 to 15 years and depressive symptoms at 15 years in both genders [12]. Andersen et al. [13], however, found that decreased intake of fruit and vegetables, over a 3-year follow-up period of 15–18-year-olds, was associated with an increased risk of reporting depressive symptoms, even after adjusting for parents’ education level, household income, baseline depressive symptoms, gender and lifestyle changes.

There is a global concern about FV, with higher prevalence of low FV generally among those more deprived [14]. In children and adolescents, key correlates of low FV include gender, age, socioeconomic position, preferences, parental intake, and home availability/accessibility [15]. In the UK, only 8% of children aged 11–18 years meet the recommendation of eating five or more portions of fruit and vegetables per day [16]. A recent report highlighted the importance of ethnicity, parenting, and frequency of family activities on adolescent FV [17]. Black Caribbeans, Black Africans, and Pakistanis/Bangladeshis were more likely to consume < 5 portions of fruit and vegetables per day than their White British peers. Lower parental care and lower frequency of family activities were associated with a higher likelihood of low FV. The association between socioeconomic circumstances (SEC) and FV is inconsistent [15, 17–20]. A clustering of unhealthy behaviours in children and adolescents is also suggested by some studies, with low diet quality (e.g. low FV) related to low physical activity [17, 21, 22].

The overall aim of this study was to investigate the relationship between FV and mental health in an ethnically diverse cohort of British adolescents. The specific questions addressed were: (i) is FV a longitudinal correlate of mental health across adolescence; (ii) are there any gender or ethnic variations in any observed associations; (iii) are any of the associations in (i) and (ii) accounted for by differences in family and socioeconomic environments?

## Methods

### Design and sample

Details of the Determinants of Adolescent (now young Adult) Social wellbeing and Health (DASH) cohort study can be found on a website [23] and in a published cohort profile [24]. In 2002–03, a total of 6643 pupils aged 11–13 years, from 51 secondary schools in 10 London boroughs, enrolled at baseline. The baseline sample was recruited from schools in the London boroughs of Brent, Croydon, Hackney, Hammersmith & Fulham, Haringey, Lambeth, Newham, Southwark, Waltham Forest and Wandsworth. These boroughs were selected as they have high proportions and numbers of people from ethnic minority groups. Schools with at least 5% of people of Black Caribbean descent were identified using school censuses provided by the Department of Education and Skills [24]. Within each borough, schools were selected to enable representation at, above and below the national averages for academic performance based on reports from the Office for Standards in Education [24]. The classes were randomly selected and were all mixed ability classes. In 2005–06, 4779 of the pupils, from 49 schools, participated in the follow-up study at the age of 14–16 years, with the mean follow-up time of 2.62 years (standard deviation 0.22). Two schools did not participate in the follow-up study, one due to space restrictions during building renovations and another due to the pressures of examination timetables [24]. The response rate was 88% at baseline and 72% at follow-up. A total of 4683 pupils were included in the analysis after excluding participants with missing data in mental health measurements at either baseline or follow-up (*n* = 96). Data were collected using self-complete questionnaires and pupils were supervised by trained field assistants.

### Outcome

Mental health was assessed using the 25-item self-report Strengths and Difficulties Questionnaire (SDQ) [25], which has been validated in ethnically diverse samples [26, 27]. It comprises of five subscales of five items each rated on a three-point scale, which, respectively, represent five relevant dimensions: emotional symptoms, conduct problems, hyperactivity, peer problems, and prosocial behaviour. A Total Difficulties Score (TDS), ranging from 0 to 40, was derived by adding up scores from the first four of these subscales, with a higher score indicating more psychological distress. A cut-off of TDS > 17 was used to identify probable clinical cases of mental disorders, based on validation approach in national data where approximately 10% of adolescents had scores within this band [28–30].

### Explanatory variables and confounders

Intake of fruit and vegetable was assessed separately using validated questions in national surveys [31], which had been previously used in measuring FV in adolescents [32, 33]. Fruit intake was measured with the question ‘How many portions of fruit do you usually eat in a day?’ Response categories included ‘5 or more portions per day’, ‘4 portions per day’, ‘3 portions per day’, ‘2 portions per day’, ‘1 portion per day’, ‘Eat some days but not every day’, and ‘Never eat’. Vegetable intake was measured with a similar question ‘How many portions of vegetables do you usually eat in a day?’, and the response categories were the same as for fruit. Examples of one portion (e.g. a handful of carrots, an apple, or a bowl of fruit/vegetable salad) were given along with the questions for more accurate estimation of the portion size. Total FV was derived by summing the reported portions of fruit and vegetables consumed daily, which was further collapsed into ‘≥5 portions/day’, ‘1–4 portions/day’, and ‘< 1 portion/day’, respectively representing recommended or more intake according to national guidelines, moderately low, and very low intake [34].

Other information used were demographics (age, gender, and ethnicity), own lifestyles (physical activity, current smoking, current alcohol consumption, special diet, and diet-related anxiety), parental lifestyles (paternal smoking, maternal smoking, paternal overweight, and maternal overweight), parenting (perceived parental care and parental control [35]), and SEC (family affluence [36]). Age was determined from the reported date of birth. Ethnicity was self-defined and checked against reported parental ethnicity and grandparents’ country of birth. The Bangladeshi and Pakistani ethnic groups were combined due to small sample sizes. Physical activity, based on 37 vigorous sporting activities (e.g. running, cycling, football, kick-boxing) and the frequency of taking part in each activity (every day, most days, weekly, less than weekly, and never) [34], was classified into the number of activities taken per week and coded into five categories: ‘≥5 times/week’, ‘3–4 times/week’, ‘twice/week’, ‘once/week’, and ‘none’. Binary responses (‘Yes’ or ‘No’) were created for special diets (vegetarians, religious prohibition of food or slimming diets), diet-related anxiety (worried about weight gain or unhappy if overeating), current smoking, current alcohol consumption, parental smoking, and parental overweight. Parental care and control were measured using the eight-item Parental Bonding Instrument [35], with scores categorised as ‘low’ (care/control< 14), ‘medium’ (care/control = 14–15) and ‘high’ (care/control = 16) based on thresholds for tertiles at age 11–13 years. Family affluence was measured using the Family Affluence Scale (FAS) [36], derived by summing the number of cars/vans, computers, and holidays, categorised as ‘high’ (FAS ≥ 3), ‘medium’ (FAS = 1–2) and ‘low’ (FAS = 0). Multidimensional measures such as this are known to better capture disadvantage in ethnic minorities than traditional measures such as occupational class [24, 37], and it correlates well with parental employment status [38].

### Statistical analysis

Data analyses for this study were conducted with STATA 13.0 (Stata Corp., College Station, TX, USA). Missing data in each categorical variable were recoded as ‘not stated’. A three-level random intercept model was used to explore the association between FV and mean TDS across adolescence, as there were repeated measures (Level 1) which were obtained from the same pupil (Level 2) at 11–13 years and 14–16 years, respectively, with pupils clustered within 49 schools (Level 3). All variables were considered as time (age)-dependent except gender and ethnicity.

As data used in the analysis were collected at two timepoints (2002–03 and 2005–06), the effect of age fitted as a quadratic or cubic function could not be tested. Models included the linear effect of age (grand-mean centred, in years). TDS was initially regressed on FV only (Model 1), and adjustments were sequentially undertaken with each variable added singly. Families of models were presented, and any specific effects were noted in the text. Model 2 refers to additional adjustments for age, gender and ethnicity. Model 3 refers to additional adjustments for own lifestyles (physical activity, current smoking, current alcohol consumption, special diet, and diet-related anxiety). Model 4 refers to additional adjustments for family factors (paternal smoking, maternal smoking, paternal overweight, maternal overweight, parental care, and parental control) and SEC. To ensure the parsimony of the final model (Model 5), only variables with *P* < 0.05 in Model 4 based on the Wald test were included. The association between FV and probable clinical cases (TDS > 17) across adolescence was examined using the three-level mixed-effects logistic regression with random intercepts. The model building approach corresponded with that described for mean TDS. Interactions including FV × gender, FV × ethnicity, FV × parental care, FV × parental control, and FV × family affluence were tested in the simple model for both mean TDS and probable clinical cases and turned out to be not statistically significant, suggesting that the associations observed with FV did not vary across these variables.

## Results

### Sample characteristics

Table 1 gives a description of the sample at 11–13 years and 14–16 years by gender and ethnicity (see full tables on Additional files 1 and 2). Compared with those aged 11–13 years, adolescents aged 14–16 years had a lower mean TDS and a lower proportion of probable clinical cases. There were significant variations in FV by ethnicity and age. At 11–13 years, Black Africans were less likely to consume ≥5 portions/day and more likely to consume < 1 portion/day than their White peers. At 14–16 years, this pattern was observed for most ethnic minority groups except Indians.

**Table 1:** Key sample characteristics by age, gender and ethnicity, presented as *n* (%)

	White British		Black Caribbean		Black African		Indian		Pakistani/Bangladeshi		All	
	Males (*N* = 484)	Females (*N* = 383)	Males (*N* = 344)	Females (*N* = 351)	Males (*N* = 372)	Females (*N* = 446)	Males (*N* = 224)	Females (*N* = 172)	Males (*N* = 310)	Females (*N* = 141)	Males (*N* = 2548)	Females (*N* = 2135)
TDS at 11–13 years [as mean (SD)]	11.3 (5.0)	11.1 (5.1)	10.8 (5.1)	11.4 (5.2)	9.6^a^ (4.6)	11.1 (5.3)	10.6 (5.8)	9.4^a^ (4.6)	10.2^a^ (5.1)	11.3 (5.4)	10.6 (5.1)	11.1 (5.2)
TDS at 14–16 years [as mean (SD)]	10.3^b^ (4.6)	11.4 (4.9)	9.6^a^/^b^ (4.6)	11.0 (4.9)	9.0^a^ (4.5)	10.9 (4.9)	9.7 (5.2)	9.7^a^ (4.5)	9.5^a^ (4.5)	11.0 (5.0)	9.7^b^ (4.6)	11.1 (4.9)
TDS > 17 at 11–13 years	55 (11.4)	45 (11.8)	32 (9.3)	44 (12.5)	22 (5.9^a^)	52 (11.7)	30 (13.4)	11 (6.4)	26 (8.4)	17 (12.1)	246 (9.7)	252 (11.8)
TDS > 17 at 14–16 years	33 (6.8)	41 (10.7)	20 (5.8)	36 (10.3)	15 (4.0)	48 (10.8)	15 (6.7)	8 (4.7^a^)	13 (4.2)	18 (12.8)	147 (5.8)	224 (10.5)
Fruit and vegetable consumption at 11–13 years												
≥ 5 portions/day	166 (34.3)	118 (30.8)	98 (28.5)	96 (27.4)	90 (24.2^a^)	106 (23.8^a^)	87 (38.8)	62 (36.1)	78 (25.2^a^)	39 (27.7)	801 (31.4)	632 (29.6)
1–4 portions/day	189 (39.1)	156 (40.7)	123 (35.8)	132 (37.6)	125 (33.6)	145 (32.5^a^)	76 (33.9)	66 (38.4)	109 (35.2)	57 (40.4)	882 (34.6)	785 (36.8)
< 1 portion/day	87 (18.0)	84 (21.9)	58 (16.9)	96 (27.4)	89 (23.9^a^)	128 (28.7^a^)	28 (12.5)	23 (13.4^a^)	79 (25.5^a^)	28 (19.9)	476 (18.7)	473 (22.2)
Not stated	42 (8.7)	25 (6.5)	65 (18.9^a^)	27 (7.7)	68 (18.3^a^)	67 (15.0^a^)	33 (14.7^a^)	21 (12.2^a^)	44 (14.2^a^)	17 (12.1^a^)	389 (15.3)	245 (11.5)
Fruit and vegetable consumption at 14–16 years												
≥ 5 portions/day	178 (36.8)	151 (39.4)	93 (27.0^a^)	95 (27.1^a^)	92 (24.7^a^)	105 (23.5^a^)	80 (35.7)	65 (37.8)	73 (23.6^a^)	36 (25.5^a^)	778 (30.5)	693 (32.5)
1–4 portions/day	216 (44.6)	149 (38.9)	138 (40.1)	127 (36.2)	145 (39.0)	169 (37.9)	113 (50.5^b^)	80 (46.5)	137 (44.2)	58 (41.1)	1100 (43.2)	823 (38.6)
< 1 portion/day	89 (18.4)	80 (20.9)	110 (32.0^a^/^b^)	127 (36.2^a^)	134 (36.0^a^/^b^)	171 (38.3^a^/^b^)	30 (13.4)	27 (15.7)	97 (31.3^a^)	47 (33.3^a^)	659 (25.9)	610 (28.6)
Not stated	1 (0.2^b^)	3 (0.8^b^)	3 (0.9^b^)	2 (0.6^b^)	1 (0.3^b^)	1 (0.2^b^)	1 (0.4^b^)	0^a^/^b^	3 (1.0^b^)	0^a^/^b^	11 (0.4)	9 (0.4)
Parental care at 11–13 years												
High	181 (37.4)	168 (43.9)	129 (37.5)	137 (39.0)	134 (36.0)	149 (33.4^a^)	92 (41.1)	67 (39.0)	121 (39.0)	54 (38.3)	977 (38.3)	805 (37.7)
Medium	163 (33.7)	120 (31.3)	80 (23.3^a^)	85 (24.2)	91 (24.5^a^)	121 (27.1)	54 (24.1)	52 (30.2)	91 (29.4)	39 (27.7)	697 (27.4)	605 (28.3)
Low	125 (25.8)	83 (21.7)	105 (30.5)	105 (29.9)	117 (31.5)	149 (33.4^a^)	66 (29.5)	49 (28.5)	88 (28.4)	44 (31.2)	722 (28.3)	612 (28.7)
Not stated	15 (3.1)	12 (3.1)	30 (8.7^a^)	24 (6.8)	30 (8.1^a^)	27 (6.1)	12 (5.4)	4 (2.3)	10 (3.2)	4 (2.8)	152 (6.0)	113 (5.3)
Parental care at 14–16 years												
High	124 (25.6^b^)	93 (24.3^b^)	81 (23.6^b^)	55 (15.7^a^/^b^)	85 (22.8^b^)	73 (16.4^a^/^b^)	67 (29.9)	43 (25.0)	88 (28.4)	37 (26.2)	653 (25.6)	434 (20.3)
Medium	141 (29.1)	111 (29.0)	92 (26.7)	97 (27.6)	101 (27.2)	106 (23.8)	71 (31.7)	48 (27.9)	87 (28.1)	30 (21.3)	738 (29.0)	544 (25.5)
Low	217 (44.8^b^)	175 (45.7^b^)	165 (48.0^b^)	196 (55.8^b^)	177 (47.6^b^)	260 (58.3^a^/^b^)	85 (38.0)	80 (46.5^b^)	131 (42.3^b^)	73 (51.8^b^)	1126 (44.2)	1134 (53.1)
Not stated	2 (0.4^b^)	4 (1.0)	6 (1.7^b^)	3 (0.9^b^)	9 (2.4^b^)	7 (1.6^b^)	1 (0.4)	1 (0.6)	4 (1.3)	1 (0.7)	31 (1.2)	23 (1.1)
Paternal control at 11–13 years												
Low	169 (34.9)	163 (42.6)	109 (31.7)	80 (22.8^a^)	75 (20.2^a^)	86 (19.3^a^)	51 (22.8^a^)	38 (22.1^a^)	55 (17.7^a^)	31 (22.0^a^)	658 (25.8)	551 (25.8)
Medium	193 (39.9)	142 (37.1)	109 (31.7)	125 (35.6)	130 (35.0)	152 (34.1)	75 (33.5)	70 (40.7)	122 (39.4)	47 (33.3)	941 (36.9)	760 (35.6)
High	104 (21.5)	68 (17.8)	97 (28.2)	126 (35.9^a^)	137 (36.8^a^)	178 (39.9^a^)	86 (38.4^a^)	59 (34.3^a^)	123 (39.7^a^)	60 (42.6^a^)	787 (30.9)	710 (33.3)
Not stated	18 (3.7)	10 (2.6)	29 (8.4^a^)	20 (5.7)	30 (8.1)	30 (6.7)	12 (5.4)	5 (2.9)	10 (3.2)	3 (2.1)	162 (6.4)	114 (5.3)
Paternal control at 14–16 years												
Low	217 (44.8^b^)	166 (43.3)	114 (33.1^a^)	91 (25.9^a^)	91 (24.5^a^)	102 (22.9^a^)	65 (29.0^a^)	38 (22.1^a^)	69 (22.3^a^)	30 (21.3^a^)	818 (32.1)	591 (27.7)
Medium	170 (35.1)	113 (29.5)	117 (34.0)	109 (31.1)	138 (37.1)	128 (28.7)	78 (34.8)	56 (32.6)	122 (39.4)	43 (30.5)	915 (35.9)	658 (30.8)
High	95 (19.6)	99 (25.8)	106 (30.8^a^)	148 (42.2^a^)	132 (35.5^a^)	208 (46.6^a^)	79 (35.3^a^)	78 (45.3^a^)	115 (37.1^a^)	68 (48.2^a^)	782 (30.7)	863 (40.4)
Not stated	2 (0.4^b^)	5 (1.3)	7 (2.0^b^)	3 (0.9^b^)	11 (3.0^a^/^b^)	8 (1.8^b^)	2 (0.9)	0^a^/^b^	4 (1.3)	0^a^/^b^	33 (1.3)	23 (1.1)
Family affluence at 11–13 years												
High	306 (63.2)	243 (63.4)	169 (49.1^a^)	153 (43.6^a^)	196 (52.7^a^)	232 (52.0^a^)	124 (55.4)	90 (52.3)	175 (56.5)	55 (39.0^a^)	1382 (54.2)	1081 (50.6)
Medium	114 (23.6)	93 (24.3)	98 (28.5)	123 (35.0^a^)	95 (25.5)	136 (30.5)	52 (23.2)	53 (30.8)	82 (26.5)	61 (43.3^a^)	672 (26.4)	673 (31.5)
Low	21 (4.3)	16 (4.2)	15 (4.4)	21 (6.0)	11 (3.0)	12 (2.7)	5 (2.2)	3 (1.7)	10 (3.2)	5 (3.5)	101 (4.0)	88 (4.1)
Not stated	43 (8.9)	31 (8.1)	62 (18.0^a^)	54 (15.4^a^)	70 (18.8^a^)	66 (14.8^a^)	43 (19.2^a^)	26 (15.1^a^)	43 (13.9^a^)	20 (14.2^a^)	393 (15.4)	293 (13.7)
Family affluence at 14–16 years												
High	341 (70.5)	272 (71.0)	210 (61.0^a^/^b^)	202 (57.5^a^/^b^)	252 (67.7^b^)	281 (63.0^b^)	161 (71.9^b^)	123 (71.5^b^)	228 (73.5^b^)	81 (57.4^a^/^b^)	1722 (67.6)	1347 (63.1)
Medium	123 (25.4)	92 (24.0)	106 (30.8)	130 (37.0^a^)	99 (26.6)	149 (33.4^a^)	52 (23.2)	44 (25.6)	72 (23.2)	56 (39.7^a^)	702 (27.6)	691 (32.4)
Low	10 (2.1)	6 (1.6)	12 (3.5)	12 (3.4)	5 (1.3)	3 (0.7)	3 (1.3)	0^a^/^b^	3 (1.0)	0^a^/^b^	45 (1.8)	36 (1.7)
Not stated	10 (2.1^b^)	13 (3.4)	16 (4.7^b^)	7 (2.0^b^)	16 (4.3^b^)	13 (2.9^b^)	8 (3.6^a^)	5 (2.9^b^)	7 (2.3^b^)	4 (2.8^b^)	79 (3.1)	61 (2.9)

*TDS* Total Difficulties Score

^a^Indicates differences compared with White British within the same age and gender group

^b^Indicates differences compared with 11–13 years within the same gender and ethnic group

### FV and mean TDS across adolescence

Table 2 shows the association between FV and pooled mean TDS across age, unadjusted and adjusted for demographics, own lifestyles, parental lifestyles, parenting and SEC. In the univariate model (Model 1), mean TDS was higher in those who reported 1–4 portions/day (marginally) or < 1 portion/day compared with those who reported ≥5 portions/day. Additional adjustments for age, gender and ethnicity (Model 2) and own lifestyles (Model 3) did not alter these associations. Adjustments for parental lifestyles, parenting and SEC (Model 4), however, removed the statistically significant association with 1–4 portions/day and attenuated the effect of < 1 portion/day. The addition of parental care accounted for most of the reduction of the effect of both 1–4 portions/day and < 1 portion/day. Effects of FV in the parsimonious model (Model 5) were similar to those in Model 4.

**Table 2:** The association between fruit and vegetable consumption and total difficulties score from 11 to 13 years to 14–16 years

	Model 1^a^		Model 2^b^		Model 3^c^		Model 4^d^		Model 5^e^	
	Coef (95% CI)	*P*	Coef (95% CI)	*P*	Coef (95% CI)	*P*	Coef (95% CI)	*P*	Coef (95% CI)	*P*
Fixed effects										
Fruit and vegetable consumption (ref: ≥5 portions/day)										
1–4 portions/day	0.22 (0.00, 0.44)	0.054	0.26 (0.04, 0.49)	0.019	0.27 (0.05, 0.49)	0.016	0.13 (−0.09, 0.34)	0.242	0.13 (− 0.09, 0.34)	0.253
< 1 portion/day	0.77 (0.51, 1.03)	< 0.001	0.85 (0.58, 1.11)	< 0.001	0.81 (0.54, 1.07)	< 0.001	0.56 (0.30, 0.81)	< 0.001	0.55 (0.29, 0.81)	< 0.001
Not stated	0.91 (0.54, 1.28)	< 0.001	0.61 (0.22, 0.99)	0.002	−0.15 (−0.85, 0.55)	0.672	−0.40 (−1.09, 0.29)	0.254	−0.40 (−1.08, 0.28)	0.248
Age			−0.18 (− 0.23, − 0.12)	< 0.001	−0.28 (− 0.34, − 0.21)	< 0.001	−0.34 (− 0.41, − 0.27)	< 0.001	−0.34 (− 0.41, − 0.27)	< 0.001
Gender (ref: male)										
Female			0.90 (0.64, 1.16)	< 0.001	0.49 (0.23, 0.75)	< 0.001	0.34 (0.09, 0.59)	0.007	0.34 (0.09, 0.58)	0.007
Ethnicity (ref: White British)										
Black Caribbean			−0.38 (−0.81, 0.05)	0.083	−0.17 (− 0.59, 0.25)	0.425	− 0.43 (− 0.83, − 0.03)	0.037	−0.42 (− 0.82, − 0.02)	0.039
Black African			−0.97 (−1.39, − 0.55)	< 0.001	−0.53 (− 0.94, − 0.11)	0.013	−0.71 (− 1.12, − 0.30)	0.001	−0.68 (− 1.08, − 0.28)	0.001
Indian			−0.96 (− 1.48, − 0.44)	< 0.001	−0.67 (− 1.20, − 0.14)	0.014	−0.73 (− 1.24, − 0.21)	0.005	−0.62 (−1.12, − 0.13)	0.014
Pakistani/Bangladeshi			−0.74 (−1.23, − 0.24)	0.004	−0.41 (− 0.93, 0.12)	0.133	−0.63 (− 1.14, − 0.12)	0.015	−0.50 (− 0.98, 0.02)	0.043
Parental care (ref: high)										
Medium							0.64 (0.41, 0.87)	< 0.001	0.63 (0.41, 0.86)	< 0.001
Low							1.45 (1.21, 1.69)	< 0.001	1.45 (1.20, 1.69)	< 0.001
Not stated							0.39 (−0.49, 1.28)	0.382	0.40 (−0.48, 1.29)	0.375
Parental control (ref: low)										
Medium							0.41 (0.19, 0.64)	< 0.001	0.42 (0.19, 0.64)	< 0.001
High							1.67 (1.42, 1.91)	< 0.001	1.67 (1.43, 1.92)	< 0.001
Not stated							1.46 (0.58, 2.34)	0.001	1.47 (0.59, 2.34)	0.001
Family affluence (ref: high)										
Medium							0.09 (−0.12, 0.30)	0.393	0.09 (−0.12, 0.30)	0.403
Low							0.73 (0.18, 1.28)	0.009	0.73 (0.18, 1.27)	0.009
Not stated							−0.14 (−0.51, 0.23)	0.457	−0.14 (− 0.51, 0.23)	0.455
Constant	10.27 (10.04, 10.49)	< 0.001	10.28 (9.93, 10.63)	< 0.001	9.19 (8.80, 9.59)	< 0.001	7.71 (7.29, 8.14)	< 0.001	7.75 (7.33, 8.17)	< 0.001
Random effects										
Level 3 (school)										
Intercept	0.21 (0.10, 0.45)		0.10 (0.04, 0.31)		0.09 (0.03, 0.28)		0.07 (0.02, 0.26)		0.07 (0.02, 0.25)	
Level 2 (individual)										
Intercept	11.41 (10.66, 12.20)		11.21 (10.48, 12.00)		10.39 (9.68, 11.14)		8.88 (8.24, 9.58)		8.89 (8.24, 9.58)	
Residue	12.79 (12.29, 13.32)		12.70 (12.19, 13.22)		12.49 (11.99, 13.01)		12.14 (11.66, 12.65)		12.15 (11.66, 12.66)	

^a^Model 1: coefficients were estimated with linear-mixed models with random intercept, without any adjustments

^b^Model 2: same as model 1 + adjustments for age, gender and ethnicity

^c^Model 3: same as model 2 + adjustments for physical activity, current smoking, current alcohol consumption, special diet and diet-related anxiety

^d^Model 4: same as model 3 + adjustments for paternal smoking, maternal smoking, paternal overweight, maternal overweight, parental care, parental control and family affluence

^e^Model 5: based on Wald tests of variables in model 4, adjusted for age, gender, ethnicity, physical activity, current smoking, current alcohol consumption, diet-related anxiety, paternal smoking, maternal smoking, paternal overweight, maternal overweight, parental care, parental control and family affluence

**Fig. 1:**
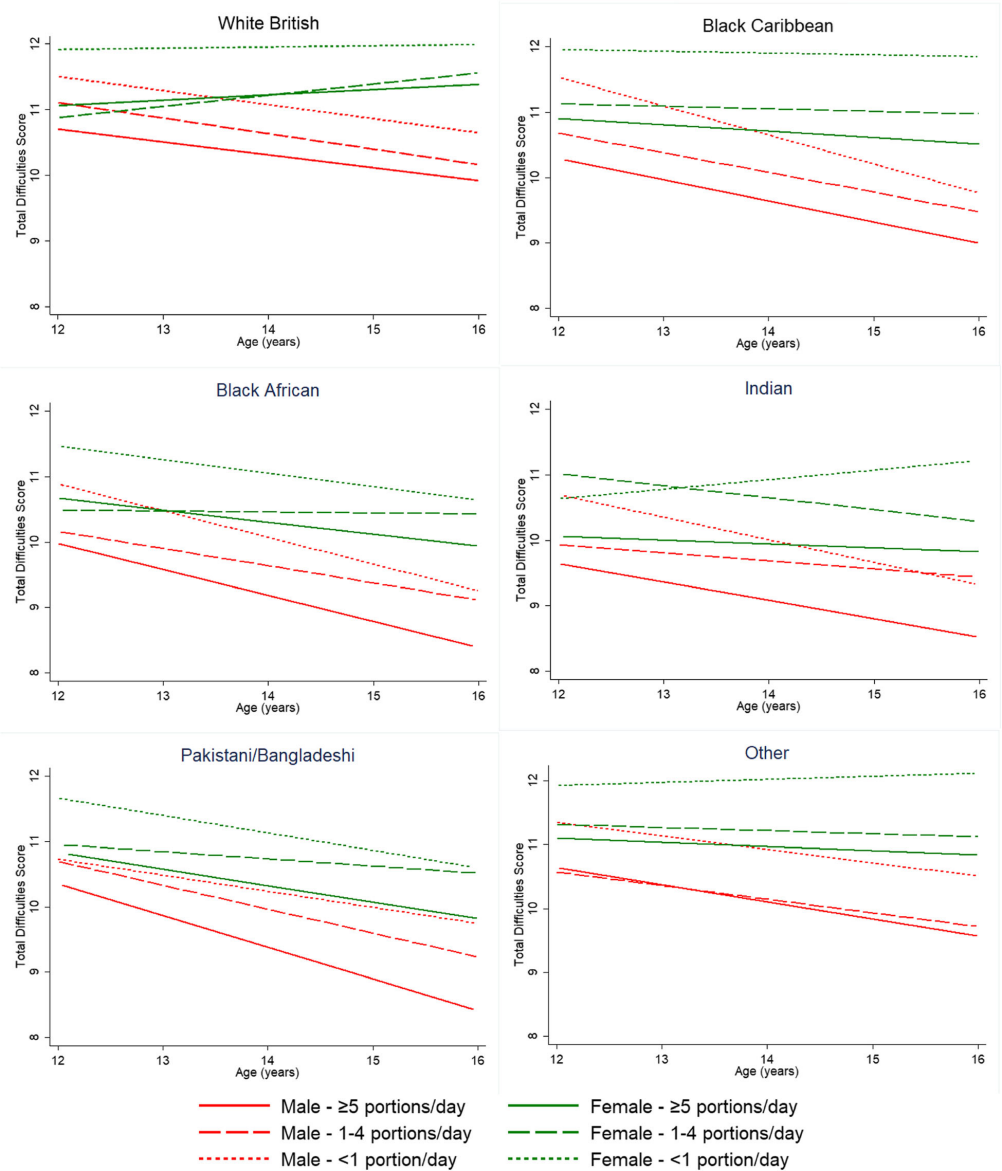
Trajectories of mean Total Difficulties Score (TDS) by fruit and vegetable consumption, gender and ethnicity from the age of 12 to 16 years. TDS means were predicted from linear-mixed models with random intercept, with adjustments for fruit and vegetable consumption, age, gender, ethnicity, physical activity, current smoking, current alcohol consumption, diet-related anxiety, paternal smoking, maternal smoking, paternal overweight, maternal overweight, parental care, parental control and family affluence. Means were restricted to 12–16 years of age, where estimations were robust

Figure 1 shows predicted mean TDS by FV, gender and ethnicity across adolescence, derived from the parsimonious model in Table 2. Within each ethnic group, mean TDS was consistently higher among those reporting < 1 portion/day than those reporting ≥5 portions/day. Differences between 1 and 4 portions/day and ≥ 5 portions/day were not consistently observed in both genders and all ethnic groups.

### FV and probable clinical cases across adolescence

Table 3 shows the association between FV and probable clinical cases (TDS > 17), pooled across the age, unadjusted and adjusted for demographics, own lifestyles, parental lifestyles, family life and SEC. In the univaritate model (Model 1), FV < 1 portion/day was associated with a higher likelihood of being a probable clinical case. Additional adjustments for age, gender and ethnicity (Model 2) and own lifestyles (Model 3) did not alter the association with < 1 portion/day. Additional adjustments for parental lifestyles, parenting and SEC (Model 4) partially attenuated the effect of < 1 portion/day. As with mean TDS, adjusting for parental care accounted for most reduction of the Odds Ratio (OR). In the parsimonious model (Model 5), the OR associated with < 1 portion/day was similar to that in Model 4. Unlike the results for mean TDS, 1–4 portions/day was not associated with probable clinical cases.

**Table 3:** The association between fruit and vegetable consumption and probable clinical cases (total difficulties score > 17) from 11 to 13 years to 14–16 years

	Model 1^a^		Model 2^b^		Model 3^c^		Model 4^d^		Model 5^e^	
	OR (95% CI)	*P*	OR (95% CI)	*P*	OR (95% CI)	*P*	OR (95% CI)	*P*	OR (95% CI)	*P*
Fixed effects										
Fruit and vegetable consumption (ref: ≥5 portions/day)										
1–4 portions/day	1.08 (0.86, 1.35)	0.498	1.12 (0.90–1.41)	0.315	1.14 (0.91–1.42)	0.271	1.06 (0.84–1.33)	0.631	1.06 (0.85–1.34)	0.595
< 1 portion/day	1.51 (1.18, 1.94)	0.001	1.62 (1.26–2.09)	< 0.001	1.62 (1.25–2.09)	< 0.001	1.42 (1.10–1.84)	0.008	1.43 (1.11–1.85)	0.007
Not stated	1.74 (1.22, 2.47)	0.002	1.44 (1.00–2.07)	0.049	1.80 (0.92–3.51)	0.084	1.69 (0.86–3.34)	0.130	1.72 (0.88–3.38)	0.112
Age			0.85 (0.80–0.90)	< 0.001	0.81 (0.76–0.88)	< 0.001	0.78 (0.73–0.85)	< 0.001	0.78 (0.73–0.84)	< 0.001
Gender (ref: male)										
Female			1.70 (1.38–2.09)	< 0.001	1.36 (1.10–1.67)	0.004	1.26 (1.02–1.55)	0.034	1.26 (1.02–1.56)	0.029
Ethnicity (ref: White British)										
Black Caribbean			0.85 (0.60–1.21)	0.370	0.96 (0.68–1.35)	0.793	0.78 (0.55–1.11)	0.171	0.77 (0.54–1.09)	0.144
Black African			0.68 (0.48–0.96)	0.026	0.78 (0.55–1.11)	0.167	0.67 (0.46–0.96)	0.030	0.63 (0.44–0.90)	0.011
Indian			0.75 (0.49–1.15)	0.189	0.73 (0.46–1.14)	0.161	0.68 (0.43–1.07)	0.098	0.64 (0.41–1.01)	0.053
Pakistani/Bangladeshi			0.76 (0.50–1.14)	0.186	0.68 (0.43–1.06)	0.091	0.57 (0.36–0.89)	0.015	0.54 (0.34–0.84)	0.007
Parental care (ref: high)										
Medium							1.23 (0.94–1.60)	0.129	1.22 (0.94–1.59)	0.140
Low							2.07 (1.60–2.68)	< 0.001	2.07 (1.60–2.67)	< 0.001
Not stated							0.82 (0.34–2.02)	0.673	0.81 (0.33–1.97)	0.637
Parental control (ref: low)										
Medium							1.01 (0.77–1.32)	0.961	1.00 (0.77–1.31)	0.984
High							2.54 (1.96–3.30)	< 0.001	2.54 (1.95–3.29)	< 0.001
Not stated							2.65 (1.14–6.16)	0.024	2.62 (1.13–6.08)	0.025
Family affluence (ref: high)										
Medium							1.17 (0.95–1.45)	0.138		
Low							1.15 (0.69–1.93)	0.583		
Not stated							1.05 (0.73–1.53)	0.783		
Constant	0.03 (0.02, 0.04)	< 0.001	0.027 (0.019, 0.039)	< 0.001	0.018 (0.012, 0.027)	< 0.001	0.008 (0.005, 0.014)	< 0.001	0.009 (0.006, 0.015)	< 0.001
Random effects										
Level 3 (school)										
Intercept	0.01 (0.00, 10.43)		0.000		0.000		0.000		0.000	
Level 3 (school) > Level 2 (individual)										
Intercept	3.13 (2.42, 4.07)		3.04 (2.34, 3.95)		2.68 (2.04, 3.51)		2.48 (1.87, 3.29)		2.48 (1.87, 3.29)	

^a^Model 1: odds ratios were estimated with mixed-effects logistic regression with random intercept, without any adjustments

^b^Model 2: same as model 1 + adjustments for age, gender and ethnicity

^c^Model 3: same as model 2 + adjustments for physical activity, current smoking, current alcohol consumption, special diet and diet-related anxiety

^d^Model 4: same as model 3 + adjustments for paternal smoking, maternal smoking, paternal overweight, maternal overweight, parental care, parental control and family affluence

^e^Model 5: based on Wald tests of variables in model 4, adjusted for age, gender, ethnicity, physical activity, current smoking, current alcohol consumption, special diet, diet-related anxiety, paternal smoking, paternal overweight, maternal overweight, parental care and parental control

## Discussion

### Principal findings

Low FV was common among adolescents, with approximately 60–70% of adolescents reporting < 5 portions/day and 20–30% reporting < 1 portion/day. In late adolescence, most ethnic minority groups reported lower FV than their White peers. Very low intake was an independent longitudinal correlate of a higher TDS and a higher likelihood of being a probable clinical case across adolescence. These associations did not vary by gender or ethnicity. Low parental care accounted for part of the association between FV and mental health.

### Comparisons with other studies

Findings in the present study are generally consistent with those in prospective observational and intervention studies of adults with various lengths of follow-up, which have shown that FV is beneficial to mental health [39–42]. It also adds to the sparse evidence for young people, namely three longitudinal studies with similarly large samples in different contexts (Canada, Australia, and Denmark) that have shown mixed results [11–13]. Other studies about diet and mental health focused on diet quality or dietary patterns as the exposure of interest. A systematic review including 12 epidemiological studies (9 cross-sectional, 3 prospective) found inconsistent trends for the relationships between healthy diet patterns or quality and better mental health in children and adolescents, suggesting a limited level of evidence [43]. In another systematic review in 2017, Khalid et al. also found contradictions in the evidence for the association between healthy dietary patterns or consuming a high-quality diet and lower levels of depression or better mental health [9]. Since FV is widely regarded as an important component of healthy dietary patterns and indicator of diet quality, results from these studies also suggest the current lack of evidence to support a FV-mental health association in young people.

The absence of gender differences in the FV-mental health association is contrary to what has been reported in a prospective observational study of adults, in which Nguyen et al. suggested that the different responses between males and females might be a result of a true but unclear gender-specific mechanism, or simply due to the more reporting accuracy for FV in women [40]. Since no other studies examining gender differences in the FV-mental health association, and due to variations in the study population and methods between their study and the DASH, it remains unclear whether the inconsistent results regarding gender differences in the association was due to an age-dependent gender-specific mechanism or caused by the heterogeneity existing between two studies. Further investigations are thus warranted. To our knowledge, only one study examined ethnic-specific effects of FV on mental health. A cross-sectional study of older adults in New York City showed no associations between FV and mental health measured by Health-Related Quality of Life across Blacks, Hispanics and Chinese [44]. The lack of gender- and ethnic-specific effects in the FV-mental health association found in the present study suggests that the mechanism may be universal in adolescence, and that contextual drivers (e.g. family environments) are important.

Parental care, independent of ethnicity or SEC, had an important influence on the FV-mental health association and aligns with findings of the influence of psychosocial support in two studies, which tested the impact of social support in adults [45] or parental conflict and family social support in adolescents [32]. Findings from DASH have consistently shown that parenting and family connectedness were impactful influences on health behaviours and mental health and that this endured across adolescence and early adulthood. For example, parental care and family engagement activities are longitudinal correlates of FV [17], and higher parental care, lower parental control and more frequent family activities are associated with better mental health in adolescence regardless of ethnicity [46, 47]. Family activities were not included in the present study due to the collinearity with parental care. In similar models reported here, the adjustment for family activities instead of parental care had a similar major attenuating effect (for those with FV < 1 portion/day, mean TDS without any adjustments: Coef 0.77, 95% Confidence Interval 0.51–1.03; in the final model with family activities: Coef 0.60, 95% Confidence Interval 0.35–0.86).

The biological pathway through which FV may affect mental health remains elusive. Rooney et al. proposed several plausible mechanisms in a review: certain nutrients that fruit and vegetables contain, such as complex carbohydrates, folate, vitamin B_6_, some antioxidants and minerals, may have positive effects on mental health by modulating neurotransmitter synthesis or defending against oxidative stress and inflammation [10]. Specifically, dietary polyphenols, widely presented in fruit and vegetables, may play an important role in mental health. In addition to their well-known benefits for physical health, such as cardiovascular health [48], there is emerging evidence suggesting that polyphenols’ antioxidant properties and biomodulating effects on specific cellular signalling pathways related to synaptic plasticity and neuronal stability may render them protective against psychiatric disorders [49].

Other dietary factors that were not unadjusted for in the present study, such as meal regularity and intakes of other food items and nutrients, may have also contributed to the observed association between FV and mental health. High FV is a proxy of breakfast regularity [34] and an important indicator of healthy dietary patterns [50, 51]. Irregular breakfast consumption is a correlate of poor mental health [52, 53]. Nutrients contained in healthy foods, such as *n*-3 polyunsaturated fatty acids, B vitamins, and vitamin D, have also been suggested to be beneficial to individuals with mental health problems [54–56]. In addition, highly influenced by diet [57], gut microbiota have been shown to participate in the modulation of mental health through the microbiome-gut-brain axis [58]. There has been evidence suggesting that perturbations of gut microbiota stability and diversity during critical windows, such as prenatal, early postnatal, and adolescence phases, may lead to adverse mental health outcomes in later life [59].

### Strengths and limitations

The DASH study is the largest longitudinal study of ethnically diverse young people in the UK designed to examine ethnic inequalities in health. Self-ascribed ethnicity was compared with ethnicity of parents and grandparents to check for inconsistencies. Unlike most other studies that examined FV among young people, the sample is well characterised in relation to diversity and psychosocial measures, including parent-child relationships and multidimensional measures of socioeconomic disadvantage. Participant and item response rates were also very high, aided by enormous community support and regular updated training of research assistants during the data collection period. A limitation is a lack of detailed dietary data in adolescence due to time constraints in a large multi-purpose study which required about two days per school, and therefore, the potential confounding by other dietary components, dietary patterns or overall diet quality cannot be ruled out. As ethnic minority children tend to maintain traditional eating habits, it is also possible that they may have underestimated the quantity of vegetables they consumed per day given the composition of meals such as curries, stews, and stir-fries, which are normally traditional foods for some ethnic minority groups [32, 60]. Potential biological pathways also cannot be examined as blood samples were not collected in adolescence. The pilot study indicated that this would have incurred a significant drop in response rates [24].

### Implications for policy and practice

The findings of the present study signal that interventions to improve FV should engage with the cultural complexity of young people’s lives in urban settings. London, like many global cities, is characterised by a multiplicity of ethnicities, languages, cultures, food choices, and religious beliefs [17]. Ethnic differences in parent-child relationships, such as more time spent on family activities, more parental control and less parental care, and exposure to greater socioeconomic disadvantage than Whites [46, 47, 53] pose opportunities and also challenges to promote FV. Additionally, children and families perceive their school and neighbourhood environments to influence their intentions to maintain a healthy diet [17, 60]. Given the importance of the family as a social determinant of health and development [46, 47, 53, 61], interventions that engage with the sociocultural influences to promote FV could lever substantial benefits.

## Conclusions

Compared with recommended FV of 5 or more portions/day, very low FV (< 1 portion/day) was associated with poorer mental health across adolescence, regardless of gender or ethnicity. Parenting played an important role in this association, suggesting the importance of engaging with the cultural complexity of family lives of young people in urban environments.

## Additional files

Additional file 1: **Table S1.** Sample characteristics at 11–13 years by ethnicity and gender, presented as *n* (%). (DOCX 63 kb)

Additional file 2: **Table S2.** Sample characteristics at 14–16 years by ethnicity and gender, presented as *n* (%). (DOCX 65 kb)

## Abbreviations

DASH: Determinants of Adolescents (now young Adult) Social well-being and Health study
FAS: Family Affluence Scale
FV: fruit and vegetable consumption
OR: Odds Ratio
SDQ: Strengths and Difficulties Questionnaire
SEC: Socioeconomic Circumstances
TDS: Total Difficulties Score
UK: United Kingdom
