# Supplementary material for: Fruit and vegetable consumption and mental health across adolescence: evidence from a diverse urban British cohort study
Source: Int J Behav Nutr Phys Act. 2019 Feb 8;16:19. doi: 10.1186/s12966-019-0780-y (PMC6368762; doi:10.1186/s12966-019-0780-y)
Supplement: Supplementary file 1 — Table S1. Sample characteristics at 11–13 years by ethnicity and gender, presented as n (%). (DOCX 63 kb) [file 12966_2019_780_MOESM1_ESM.docx]

**Table S1.** Sample characteristics at 11-13 years by ethnicity and gender, presented as n (%).

|  | White British | | Black Caribbean | | Black African | | Indian | | Pakistani/Bangladeshi | | Other | |
| --- | --- | --- | --- | --- | --- | --- | --- | --- | --- | --- | --- | --- |
|  | **Males (N=484)** | **Females (N=383)** | **Males (N=344)** | **Females (N=351)** | **Males (N=372)** | **Females (N=446)** | **Males (N=224)** | **Females (N=172)** | **Males (N=310)** | **Females (N=141)** | **Males (N=814)** | **Females (N=642)** |
| TDS [mean (SD)] | 11.3  (5.0) | 11.1  (5.1) | 10.8  (5.1) | 11.4  (5.2) | 9.6*  (4.6) | 11.1  (5.3) | 10.6  (5.8) | 9.4*  (4.6) | 10.2*  (5.1) | 11.3  (5.4) | 10.8  (4.9) | 11.3  (5.2) |
| TDS >17 | 55  (11.4) | 45  (11.8) | 32  (9.3) | 44  (12.5) | 22  (5.9*) | 52  (11.7) | 30  (13.4) | 11  (6.4) | 26  (8.4) | 17  (12.1) | 81  (10.0) | 83  (12.9) |
| Fruit and vegetable consumption | | | | | | | | | | | | |
| ≥5 portions/day | 166  (34.3) | 118  (30.8) | 98  (28.5) | 96  (27.4) | 90  (24.2*) | 106  (23.8*) | 87  (38.8) | 62  (36.1) | 78  (25.2*) | 39  (27.7) | 282  (34.6) | 211  (32.9) |
| 1-4 portions/day | 189  (39.1) | 156  (40.7) | 123  (35.8) | 132  (37.6) | 125  (33.6) | 145  (32.5*) | 76  (33.9) | 66  (38.4) | 109  (35.2) | 57  (40.4) | 260  (31.9*) | 229  (35.7) |
| <1 portion/day | 87  (18.0) | 84  (21.9) | 58  (16.9) | 96  (27.4) | 89  (23.9*) | 128  (28.7*) | 28  (12.5) | 23  (13.4*) | 79  (25.5*) | 28  (19.9) | 135  (16.6) | 114  (17.8) |
| Not stated | 42  (8.7) | 25  (6.5) | 65  (18.9*) | 27  (7.7) | 68  (18.3*) | 67  (15.0*) | 33  (14.7*) | 21  (12.2*) | 44  (14.2*) | 17  (12.1*) | 137  (16.8*) | 88  (13.7*) |
| Physical activity |  |  |  |  |  |  |  |  |  |  |  |  |
| ≥5 times/week | 223  (46.1) | 135  (35.2) | 175  (50.9) | 131  (37.3) | 191  (51.3) | 141  (31.6) | 117  (52.2) | 52  (30.2) | 162  (52.3) | 50  (35.5) | 368  (45.2) | 219  (34.1) |
| 3-4 times/week | 145  (30.0) | 102  (26.6) | 77  (22.4) | 85  (24.2) | 85  (22.9) | 125  (28.0) | 53  (23.7) | 36  (20.9) | 75  (24.2) | 35  (24.8) | 199  (24.5) | 143  (22.3) |
| Twice/week | 47  (9.7) | 69  (18.0) | 23  (6.7) | 53  (15.1) | 34  (9.1) | 57  (12.8) | 18  (8.0) | 29  (16.9) | 24  (7.7) | 17  (12.1) | 67  (8.2) | 94  (14.6) |
| Once/week | 31  (6.4) | 43  (11.2) | 13  (3.8) | 47  (13.4) | 10  (2.7) | 48  (10.8) | 8  (3.6) | 23  (13.4) | 14  (4.5) | 13  (9.2) | 47  (5.8) | 82  (12.8) |
| None | 10  (2.1) | 18  (4.7) | 5  (1.5) | 14  (4.0) | 5  (1.3) | 31  (7.0) | 4  (1.8) | 22  (12.8*) | 4  (1.3) | 16  (11.4) | 18  (2.2) | 38  (5.9) |
| Not stated | 28  (5.8) | 16  (4.2) | 51  (14.8) | 21  (6.0) | 47  (12.6*) | 44  (9.9) | 24  (10.7) | 10  (5.8) | 31  (10.0) | 10  (7.1) | 115  (14.1*) | 66  (10.3*) |
| Current smoking |  |  |  |  |  |  |  |  |  |  |  |  |
| No | 413  (85.3) | 341  (89.0) | 248  (72.1*) | 292  (83.2) | 277  (74.5*) | 352  (78.9*) | 172  (76.8*) | 140  (81.4) | 252  (81.3) | 117  (83.0) | 617  (75.8*) | 512  (79.8*) |
| Yes | 9  (1.9) | 15  (3.9) | 5  (1.5) | 13  (3.7) | 1  (0.3) | 3  (0.7*) | 0* | 1  (0.6) | 4  (1.3) | 1  (0.7) | 5  (0.6) | 24  (3.7) |
| Not stated | 62  (12.8) | 27  (7.1) | 91  (26.5*) | 46  (13.1) | 94  (25.3*) | 91  (20.4*) | 52  (23.2*) | 31  (18.0*) | 54  (17.4) | 23  (16.3*) | 192  (23.6*) | 106  (16.5*) |
| Current alcohol consumption |  |  |  |  |  |  |  |  |  |  |  |  |
| No | 175  (36.2) | 184  (48.0) | 142  (41.3) | 150  (42.7) | 231  (62.1*) | 267  (59.9*) | 171  (76.3*) | 140  (81.4*) | 260  (83.9*) | 122  (86.5*) | 418  (51.4*) | 354  (55.1) |
| Yes | 248  (51.2) | 176  (46.0) | 114  (33.1*) | 150  (42.7) | 51  (13.7*) | 88  (19.7*) | 16  (7.1*) | 16  (9.3*) | 7  (2.3*) | 1  (0.7*) | 214  (26.3*) | 183  (28.5*) |
| Not stated | 61  (12.6) | 23  (6.0) | 88  (25.6*) | 51  (14.5*) | 90  (24.2*) | 91  (20.4*) | 37  (16.5) | 16  (9.3) | 43  (13.9) | 18  (12.8) | 182  (22.4*) | 105  (16.4*) |
| Special diet |  |  |  |  |  |  |  |  |  |  |  |  |
| No | 375  (77.5) | 289  (75.5) | 223  (64.8*) | 246  (70.1) | 194  (52.2*) | 255  (57.2*) | 37  (16.5*) | 38  (22.1*) | 13  (4.2*) | 3  (2.1*) | 462  (56.8*) | 385  (60.0*) |
| Yes | 67  (13.8) | 74  (19.3) | 54  (15.7) | 72  (20.5) | 125  (33.6*) | 123  (27.6*) | 170  (75.9*) | 124  (72.1*) | 269  (86.8*) | 129  (91.5*) | 245  (30.1) | 177  (27.6*) |
| Not stated | 42  (8.7) | 20  (5.2) | 67  (19.5*) | 33  (9.4) | 53  (14.2) | 68  (15.2*) | 17  (7.6) | 10  (5.8) | 28  (9.0) | 9  (6.4) | 107  (13.1) | 80  (12.5*) |
| Diet-related anxiety |  |  |  |  |  |  |  |  |  |  |  |  |
| No | 281  (58.1) | 171  (44.6) | 191  (55.5) | 162  (46.2) | 210  (56.5) | 165  (37.0) | 114  (50.9) | 67  (39.0) | 141  (45.5*) | 46  (32.6) | 437  (53.7) | 265  (41.3) |
| Yes | 160  (33.1) | 192  (50.1) | 87  (25.3) | 156  (44.4) | 108  (29.0) | 211  (47.3) | 92  (41.1) | 93  (54.1) | 138  (44.5*) | 85  (60.3) | 267  (32.8) | 297  (46.3) |
| Not stated | 43  (8.9) | 20  (5.2) | 66  (19.2*) | 33  (9.4) | 54  (14.5) | 70  (15.7*) | 18  (8.0) | 12  (7.0) | 31  (10.0) | 10  (7.1) | 110  (13.5) | 80  (12.5*) |
| Paternal smoking |  |  |  |  |  |  |  |  |  |  |  |  |
| No | 213  (44.0) | 176  (46.0) | 119  (34.6) | 80  (22.8*) | 191  (51.3) | 234  (52.5) | 162  (72.3*) | 119  (69.2*) | 175  (56.5*) | 74  (52.5) | 315  (38.7) | 232  (36.1*) |
| Yes | 156  (32.2) | 104  (27.2) | 73  (21.2*) | 80  (22.8) | 32  (8.6*) | 46  (10.3*) | 44  (19.6*) | 37  (21.5) | 87  (28.1) | 45  (31.9) | 229  (28.1) | 172  (26.8) |
| Not stated | 115  (23.8) | 103  (26.9) | 152  (44.2*) | 191  (54.4*) | 149  (40.1*) | 166  (37.2*) | 18  (8.0*) | 16  (9.3*) | 48  (15.5) | 22  (15.6*) | 270  (33.2*) | 238  (37.1*) |
| Maternal smoking |  |  |  |  |  |  |  |  |  |  |  |  |
| No | 295  (61.0) | 221  (57.7) | 234  (68.0) | 223  (63.5) | 322  (86.6*) | 390  (87.4) | 210  (93.8*) | 160  (93.0*) | 282  (91.0*) | 131  (92.9*) | 523  (64.3) | 416  (64.8) |
| Yes | 159  (32.9) | 138  (36.0) | 67  (19.5*) | 87  (24.8*) | 10  (2.7*) | 7  (1.6*) | 4  (1.8*) | 4  (2.3*) | 6  (1.9*) | 2  (1.4*) | 199  (24.5*) | 153  (23.8*) |
| Not stated | 30  (6.2) | 24  (6.3) | 43  (12.5*) | 41  (11.7) | 40  (10.8) | 49  (11.0) | 10  (4.5) | 8  (4.7) | 22  (7.1) | 8  (5.7) | 92  (11.3*) | 73  (11.4) |
| Paternal overweight |  |  |  |  |  |  |  |  |  |  |  |  |
| No | 318  (65.7) | 224  (58.5) | 184  (53.5*) | 140  (39.9*) | 210  (56.5*) | 251  (56.3) | 177  (79.0*) | 131  (76.2*) | 224  (72.3) | 92  (65.2) | 484  (59.5) | 351  (54.7) |
| Yes | 27  (5.6) | 25  (6.5) | 6  (1.7*) | 5  (1.4*) | 9  (2.4) | 12  (2.7) | 15  (6.7) | 11  (6.4) | 19  (6.1) | 12  (8.5) | 26  (3.2) | 30  (4.7) |
| Not stated | 139  (28.7) | 134  (35.0) | 154  (44.8*) | 206  (58.7*) | 153  (41.1*) | 183  (41.0) | 32  (14.3*) | 30  (17.4*) | 67  (21.6) | 37  (26.2) | 304  (37.4*) | 261  (40.7) |
| Maternal overweight |  |  |  |  |  |  |  |  |  |  |  |  |
| No | 393  (81.2) | 292  (76.2) | 272  (79.1) | 248  (70.7) | 280  (75.3) | 324  (72.6) | 186  (83.0) | 124  (72.1) | 238  (76.8) | 92  (65.2) | 622  (76.4) | 483  (75.2) |
| Yes | 26  (5.4) | 26  (6.8) | 17  (4.9) | 31  (8.8) | 28  (7.5) | 33  (7.4) | 12  (5.4) | 16  (9.3) | 27  (8.7) | 17  (12.1) | 51  (6.3) | 55  (8.6) |
| Not stated | 65  (13.4) | 65  (17.0) | 55  (16.0) | 72  (20.5) | 64  (17.2) | 89  (20.0) | 26  (11.6) | 32  (18.6) | 45  (14.5) | 32  (22.7) | 141  (17.3) | 104  (16.2) |
| Parental care |  |  |  |  |  |  |  |  |  |  |  |  |
| High | 181  (37.4) | 168  (43.9) | 129  (37.5) | 137  (39.0) | 134  (36.0) | 149  (33.4*) | 92  (41.1) | 67  (39.0) | 121  (39.0) | 54  (38.3) | 320  (39.3) | 230  (35.8) |
| Medium | 163  (33.7) | 120  (31.3) | 80  (23.3*) | 85  (24.2) | 91  (24.5*) | 121  (27.1) | 54  (24.1) | 52  (30.2) | 91  (29.4) | 39  (27.7) | 218  (26.8) | 188  (29.3) |
| Low | 125  (25.8) | 83  (21.7) | 105  (30.5) | 105  (29.9) | 117  (31.5) | 149  (33.4*) | 66  (29.5) | 49  (28.5) | 88  (28.4) | 44  (31.2) | 221  (27.1) | 182  (28.3) |
| Not stated | 15  (3.1) | 12  (3.1) | 30  (8.7*) | 24  (6.8) | 30  (8.1*) | 27  (6.1) | 12  (5.4) | 4  (2.3) | 10  (3.2) | 4  (2.8) | 55  (6.8*) | 42  (6.5) |
| Paternal control |  |  |  |  |  |  |  |  |  |  |  |  |
| Low | 169  (34.9) | 163  (42.6) | 109  (31.7) | 80  (22.8*) | 75  (20.2*) | 86  (19.3*) | 51  (22.8*) | 38  (22.1*) | 55  (17.7*) | 31  (22.0*) | 199  (24.4*) | 153  (23.8*) |
| Medium | 193  (39.9) | 142  (37.1) | 109  (31.7) | 125  (35.6) | 130  (35.0) | 152  (34.1) | 75  (33.5) | 70  (40.7) | 122  (39.4) | 47  (33.3) | 312  (38.3) | 224  (34.9) |
| High | 104  (21.5) | 68  (17.8) | 97  (28.2) | 126  (35.9*) | 137  (36.8*) | 178  (39.9*) | 86  (38.4*) | 59  (34.3*) | 123  (39.7*) | 60  (42.6*) | 240  (29.5*) | 219  (34.1*) |
| Not stated | 18  (3.7) | 10  (2.6) | 29  (8.4*) | 20  (5.7) | 30  (8.1) | 30  (6.7) | 12  (5.4) | 5  (2.9) | 10  (3.2) | 3  (2.1) | 63  (7.7*) | 46  (7.2*) |
| Family affluence |  |  |  |  |  |  |  |  |  |  |  |  |
| High | 306  (63.2) | 243  (63.4) | 169  (49.1*) | 153  (43.6*) | 196  (52.7*) | 232  (52.0*) | 124  (55.4) | 90  (52.3) | 175  (56.5) | 55  (39.0*) | 412  (50.6*) | 308  (48.0*) |
| Medium | 114  (23.6) | 93  (24.3) | 98  (28.5) | 123  (35.0*) | 95  (25.5) | 136  (30.5) | 52  (23.2) | 53  (30.8) | 82  (26.5) | 61  (43.3*) | 231  (28.4) | 207  (32.2) |
| Low | 21  (4.3) | 16  (4.2) | 15  (4.4) | 21  (6.0) | 11  (3.0) | 12  (2.7) | 5  (2.2) | 3  (1.7) | 10  (3.2) | 5  (3.5) | 39  (4.8) | 31  (4.8) |
| Not stated | 43  (8.9) | 31  (8.1) | 62  (18.0*) | 54  (15.4*) | 70  (18.8*) | 66  (14.8*) | 43  (19.2*) | 26  (15.1*) | 43  (13.9*) | 20  (14.2*) | 132  (16.2*) | 96  (15.0*) |

TDS, total difficulties score.

* indicates differences compared with White British boys/girls.
