# Supplementary material for: Fruit and vegetable consumption and mental health across adolescence: evidence from a diverse urban British cohort study
Source: Int J Behav Nutr Phys Act. 2019 Feb 8;16:19. doi: 10.1186/s12966-019-0780-y (PMC6368762; doi:10.1186/s12966-019-0780-y)
Supplement: Supplementary file 2 — Table S2. Sample characteristics at 14–16 years by ethnicity and gender, presented as n (%). (DOCX 65 kb) [file 12966_2019_780_MOESM2_ESM.docx]

**Table S2.** Sample characteristics at 14-16 years by ethnicity and gender, presented as n (%).

|  | White British | | Black Caribbean | | Black African | | Indian | | Pakistani/Bangladeshi | | Other | |
| --- | --- | --- | --- | --- | --- | --- | --- | --- | --- | --- | --- | --- |
|  | **Males (N=484)** | **Females (N=383)** | **Males (N=344)** | **Females (N=351)** | **Males (N=372)** | **Females (N=446)** | **Males (N=224)** | **Females (N=172)** | **Males (N=310)** | **Females (N=141)** | **Males (N=814)** | **Females (N=642)** |
| TDS [mean (SD)] | 10.3**  (4.6) | 11.4  (4.9) | 9.6*/**  (4.6) | 11.0  (4.9) | 9.0*  (4.5) | 10.9  (4.9) | 9.7  (5.2) | 9.7*  (4.5) | 9.5*  (4.5) | 11.0  (5.0) | 9.9**  (4.5) | 11.4  (4.8) |
| TDS >17 | 33  (6.8) | 41  (10.7) | 20  (5.8) | 36  (10.3) | 15  (4.0) | 48  (10.8) | 15  (6.7) | 8  (4.7*) | 13  (4.2) | 18  (12.8) | 51  (6.3) | 73  (11.4) |
| Fruit and vegetable consumption | | | | | | | | | | | | |
| ≥5 portions/day | 178  (36.8) | 151  (39.4) | 93  (27.0*) | 95  (27.1*) | 92  (24.7*) | 105  (23.5*) | 80  (35.7) | 65  (37.8) | 73  (23.6*) | 36  (25.5*) | 262  (32.2) | 241  (37.5) |
| 1-4 portions/day | 216  (44.6) | 149  (38.9) | 138  (40.1) | 127  (36.2) | 145  (39.0) | 169  (37.9) | 113  (50.5**) | 80  (46.5) | 137  (44.2) | 58  (41.1) | 351  (43.1**) | 240  (37.4) |
| <1 portion/day | 89  (18.4) | 80  (20.9) | 110  (32.0*/**) | 127  (36.2*) | 134  (36.0*/**) | 171  (38.3*/**) | 30  (13.4) | 27  (15.7) | 97  (31.3*) | 47  (33.3*) | 199  (24.5*/**) | 158  (24.6**) |
| Not stated | 1  (0.2**) | 3  (0.8**) | 3  (0.9**) | 2  (0.6**) | 1  (0.3**) | 1  (0.2**) | 1  (0.4**) | 0*/** | 3  (1.0**) | 0*/** | 2  (0.2**) | 3  (0.5**) |
| Physical activity |  |  |  |  |  |  |  |  |  |  |  |  |
| ≥5 times/week | 68  (14.1**) | 35  (9.1**) | 56  (16.3**) | 42  (12.0**) | 66  (17.7**) | 47  (10.5**) | 42  (18.8**) | 20  (11.6**) | 65  (21.0**) | 23  (16.3**) | 146  (17.9**) | 67  (10.4**) |
| 3-4 times/week | 194  (40.1**) | 118  (30.8) | 133  (38.7**) | 97  (27.6) | 147  (39.5**) | 130  (29.2) | 94  (42.0**) | 55  (32.0) | 104  (33.5) | 37  (26.2) | 291  (35.8**) | 161  (25.1) |
| Twice/week | 126  (26.0**) | 90  (23.5) | 81  (23.5**) | 78  (22.2) | 84  (22.6**) | 99  (22.2**) | 50  (22.3**) | 41  (23.8) | 74  (23.9**) | 35  (24.8) | 185  (22.7**) | 161  (25.1**) |
| Once/week | 73  (15.1**) | 101  (26.4**) | 57  (16.6**) | 85  (24.2**) | 54  (14.5**) | 106  (23.8**) | 31  (13.8**) | 39  (22.7) | 51  (16.5**) | 24  (17.0) | 137  (16.8**) | 165  (25.7**) |
| None | 21  (4.3) | 36  (9.4) | 13  (3.8) | 46  (13.1**) | 18  (4.8) | 63  (14.1**) | 6  (2.7) | 17  (9.9) | 13  (4.2) | 21  (14.9) | 51  (6.3**) | 81  (12.6**) |
| Not stated | 2  (0.4**) | 3  (0.8**) | 4  (1.2**) | 3  (0.9**) | 3  (0.8**) | 1  (0.2**) | 1  (0.4**) | 0*/** | 3  (1.0**) | 1  (0.7) | 4  (0.5**) | 7  (1.1**) |
| Current smoking |  |  |  |  |  |  |  |  |  |  |  |  |
| No | 400  (82.6) | 272  (71.0**) | 316  (91.9*/**) | 302  (86.0*) | 354  (95.2*/**) | 421  (94.4*/**) | 210  (93.8*/**) | 165  (95.9*/**) | 274  (88.4) | 136  (96.5*/**) | 730  (89.7*/**) | 530  (82.6*) |
| Yes | 81  (16.7**) | 108  (28.2**) | 25  (7.3*/**) | 46  (13.1*/**) | 12  (3.2*) | 22  (4.9*/**) | 13  (5.8*/**) | 7  (4.1*) | 33  (10.6**) | 4  (2.8*) | 71  (8.7*/**) | 102  (15.9*/**) |
| Not stated | 3  (0.6**) | 3  (0.8**) | 3  (0.9**) | 3  (0.9**) | 6  (1.6**) | 3  (0.7**) | 1  (0.4**) | 0*/** | 3  (1.0**) | 1  (0.7**) | 13  (1.6**) | 10  (1.6**) |
| Current alcohol consumption |  |  |  |  |  |  |  |  |  |  |  |  |
| No | 121  (25.0**) | 60  (15.7**) | 134  (39.0*) | 104  (29.6*/**) | 251  (67.5*) | 264  (59.2*) | 154  (68.8*) | 108  (62.8*/**) | 296  (95.5*/**) | 129  (91.5*) | 397  (48.8*) | 258  (40.2*/**) |
| Yes | 361  (74.6**) | 320  (83.6**) | 205  (59.6*/**) | 243  (69.2*/**) | 115  (30.9*/**) | 175  (39.2*/**) | 68  (30.4*/**) | 64  (37.2*/**) | 12  (3.9*) | 10  (7.1*) | 401  (49.3*/**) | 373  (58.1*/**) |
| Not stated | 2  (0.4**) | 3  (0.8**) | 5  (1.5**) | 4  (1.1**) | 6  (1.6**) | 7  (1.6**) | 2  (0.9**) | 0*/** | 2  (0.6**) | 2  (1.4**) | 16  (2.0**) | 11  (1.7**) |
| Special diet |  |  |  |  |  |  |  |  |  |  |  |  |
| No | 444  (91.7**) | 312  (81.5) | 291  (84.6*/**) | 282  (80.3**) | 242  (65.1*/**) | 293  (65.7*) | 44  (19.6*) | 40  (23.3*) | 20  (6.5*) | 5  (3.5*) | 559  (68.7*/**) | 430  (67.0*) |
| Yes | 39  (8.1**) | 69  (18.0) | 50  (14.5*) | 67  (19.1) | 129  (34.7*) | 152  (34.1*) | 179  (79.9*) | 132  (76.7*) | 288  (92.9*) | 136  (96.5*) | 253  (31.1*) | 209  (32.6*) |
| Not stated | 1  (0.2**) | 2  (0.5**) | 3  (0.9**) | 2  (0.6**) | 1  (0.3**) | 1  (0.2**) | 1  (0.5**) | 0*/** | 2  (0.6**) | 0*/** | 2  (0.2**) | 3  (0.5**) |
| Diet-related anxiety |  |  |  |  |  |  |  |  |  |  |  |  |
| No | 356  (73.6**) | 143  (37.3) | 252  (73.3**) | 179  (51.0*) | 294  (79.0**) | 197  (44.2) | 147  (65.6**) | 67  (39.0) | 216  (69.7**) | 48  (34.0) | 598  (73.5**) | 273  (42.5) |
| Yes | 127  (26.2) | 238  (62.1**) | 89  (25.9) | 170  (48.4*) | 77  (20.7) | 248  (55.6) | 75  (33.5) | 104  (60.5) | 91  (29.4**) | 93  (66.0) | 215  (26.4**) | 365  (56.9**) |
| Not stated | 1  (0.2**) | 2  (0.5**) | 3  (0.9**) | 2  (0.6**) | 1  (0.3**) | 1  (0.2**) | 2  (0.9**) | 1  (0.6**) | 3  (1.0**) | 0*/** | 1  (0.1**) | 4  (0.6**) |
| Paternal smoking |  |  |  |  |  |  |  |  |  |  |  |  |
| No | 259  (53.5**) | 211  (55.1) | 172  (50.0**) | 152  (43.3*/**) | 246  (66.1*/**) | 309  (69.3*/**) | 164  (73.2*) | 117  (68.0*) | 188  (60.6) | 78  (55.3) | 414  (50.9**) | 326  (50.8**) |
| Yes | 185  (38.2) | 130  (33.9) | 101  (29.4) | 120  (34.2**) | 44  (11.8*) | 57  (12.8*) | 38  (17.0*) | 45  (26.2) | 83  (26.8*) | 49  (34.8) | 277  (34.0) | 215  (33.5) |
| Not stated | 40  (8.3**) | 42  (11.0**) | 71  (20.6*/**) | 79  (22.5*/**) | 82  (22.0*/**) | 80  (17.9*/**) | 22  (9.8) | 10  (5.8) | 39  (12.6) | 14  (9.9) | 123  (15.1*/**) | 101  (15.7**) |
| Maternal smoking |  |  |  |  |  |  |  |  |  |  |  |  |
| No | 315  (65.1) | 228  (59.5) | 256  (74.4*) | 253  (72.1*) | 341  (91.7*) | 416  (93.3*/**) | 211  (94.2*) | 161  (93.6*) | 282  (91.0*) | 133  (94.3*) | 569  (69.9) | 460  (71.7*) |
| Yes | 154  (31.8) | 146  (38.1) | 64  (18.6*) | 86  (24.5*) | 13  (3.5*) | 10  (2.2*) | 3  (1.3*) | 6  (3.5*) | 8  (2.6*) | 6  (4.3*) | 214  (26.3) | 157  (24.5*) |
| Not stated | 15  (3.1) | 9  (2.4) | 24  (7.0) | 12  (3.4**) | 18  (4.8**) | 20  (4.5**) | 10  (4.5) | 5  (2.9) | 20  (6.5) | 2  (1.4) | 31  (3.8**) | 25  (3.9**) |
| Paternal overweight |  |  |  |  |  |  |  |  |  |  |  |  |
| No | 375  (77.5**) | 279  (72.8**) | 268  (77.9**) | 241  (68.7**) | 263  (70.7**) | 329  (73.8**) | 176  (78.6) | 131  (76.2) | 237  (76.5) | 107  (75.9) | 625  (76.8**) | 452  (70.4**) |
| Yes | 46  (9.5) | 42  (11.0) | 11  (3.2*) | 22  (6.3**) | 13  (3.5*) | 30  (6.7) | 20  (8.9) | 18  (10.5) | 26  (8.4) | 17  (12.1) | 50  (6.1) | 55  (8.6) |
| Not stated | 63  (13.0**) | 62  (16.2**) | 65  (18.9**) | 88  (25.1*/**) | 96  (25.8*/**) | 87  (19.5**) | 28  (12.5) | 23  (13.4) | 47  (15.2) | 17  (12.1**) | 139  (17.1**) | 135  (21.0**) |
| Maternal overweight |  |  |  |  |  |  |  |  |  |  |  |  |
| No | 396  (81.8) | 308  (80.4) | 288  (83.7) | 283  (80.6**) | 307  (82.5) | 350  (78.5) | 180  (80.4) | 130  (75.6) | 242  (78.1) | 102  (72.3) | 668  (82.1**) | 509  (79.3) |
| Yes | 38  (7.9) | 46  (12.0) | 21  (6.1) | 40  (11.4) | 23  (6.2) | 58  (13.0) | 23  (10.3) | 22  (12.8) | 28  (9.0) | 28  (19.9) | 71  (8.7) | 79  (12.3) |
| Not stated | 50  (10.3) | 29  (7.6**) | 35  (10.2) | 28  (8.0**) | 42  (11.3) | 38  (8.5**) | 21  (9.4) | 20  (11.6) | 40  (12.9) | 11  (7.8**) | 75  (9.2**) | 54  (8.4**) |
| Parental care |  |  |  |  |  |  |  |  |  |  |  |  |
| High | 124  (25.6**) | 93  (24.3**) | 81  (23.6**) | 55  (15.7*/**) | 85  (22.8**) | 73  (16.4*/**) | 67  (29.9) | 43  (25.0) | 88  (28.4) | 37  (26.2) | 208  (25.6**) | 133  (20.7**) |
| Medium | 141  (29.1) | 111  (29.0) | 92  (26.7) | 97  (27.6) | 101  (27.2) | 106  (23.8) | 71  (31.7) | 48  (27.9) | 87  (28.1) | 30  (21.3) | 246  (30.2) | 152  (23.7) |
| Low | 217  (44.8**) | 175  (45.7**) | 165  (48.0**) | 196  (55.8**) | 177  (47.6**) | 260  (58.3*/**) | 85  (38.0) | 80  (46.5**) | 131  (42.3**) | 73  (51.8**) | 351  (43.1**) | 350  (54.5**) |
| Not stated | 2  (0.4**) | 4  (1.0) | 6  (1.7**) | 3  (0.9**) | 9  (2.4**) | 7  (1.6**) | 1  (0.4) | 1  (0.6) | 4  (1.3) | 1  (0.7) | 9  (1.1**) | 7  (1.1**) |
| Paternal control |  |  |  |  |  |  |  |  |  |  |  |  |
| Low | 217  (44.8**) | 166  (43.3) | 114  (33.1*) | 91  (25.9*) | 91  (24.5*) | 102  (22.9*) | 65  (29.0*) | 38  (22.1*) | 69  (22.3*) | 30  (21.3*) | 262  (32.2*/**) | 164  (25.5*) |
| Medium | 170  (35.1) | 113  (29.5) | 117  (34.0) | 109  (31.1) | 138  (37.1) | 128  (28.7) | 78  (34.8) | 56  (32.6) | 122  (39.4) | 43  (30.5) | 290  (35.6) | 209  (32.6) |
| High | 95  (19.6) | 99  (25.8) | 106  (30.8*) | 148  (42.2*) | 132  (35.5*) | 208  (46.6*) | 79  (35.3*) | 78  (45.3*) | 115  (37.1*) | 68  (48.2*) | 255  (31.3*) | 262  (40.8)* |
| Not stated | 2  (0.4**) | 5  (1.3) | 7  (2.0**) | 3  (0.9**) | 11  (3.0*/**) | 8  (1.8**) | 2  (0.9) | 0*/** | 4  (1.3) | 0*/** | 7  (0.9**) | 7  (1.1**) |
| Family affluence |  |  |  |  |  |  |  |  |  |  |  |  |
| High | 341  (70.5) | 272  (71.0) | 210  (61.0*/**) | 202  (57.5*/**) | 252  (67.7**) | 281  (63.0**) | 161  (71.9**) | 123  (71.5**) | 228  (73.5**) | 81  (57.4*/**) | 530  (65.1**) | 388  (60.4*/**) |
| Medium | 123  (25.4) | 92  (24.0) | 106  (30.8) | 130  (37.0*) | 99  (26.6) | 149  (33.4*) | 52  (23.2) | 44  (25.6) | 72  (23.2) | 56  (39.7*) | 250  (30.7) | 220  (34.3*) |
| Low | 10  (2.1) | 6  (1.6) | 12  (3.5) | 12  (3.4) | 5  (1.3) | 3  (0.7) | 3  (1.3) | 0*/** | 3  (1.0) | 0*/** | 12  (1.5**) | 15  (2.3) |
| Not stated | 10  (2.1**) | 13  (3.4) | 16  (4.7**) | 7  (2.0**) | 16  (4.3**) | 13  (2.9**) | 8  (3.6*) | 5  (2.9**) | 7  (2.3**) | 4  (2.8**) | 22  (2.7**) | 19  (3.0**) |

TDS, total difficulties score.

* indicates differences compared with White British boys/girls.

** indicates differences compared with 11-13 years within the same gender and ethnic group.
